# Platelet distribution width correlates with prognosis of non-small cell lung cancer

**DOI:** 10.1038/s41598-017-03772-z

**Published:** 2017-06-14

**Authors:** Ming-ming Cui, Na Li, Xing Liu, Zhi-yuan Yun, Ye Niu, Yong Zhang, Boning Gao, Tiemin Liu, Rui-tao Wang

**Affiliations:** 1 Department of Internal Medicine, Harbin Medical University Cancer Hospital, Harbin Medical University, Harbin, Heilongjiang 150081 China; 2 Department of Endocrinology, Ansteel Group Hospital, AnShan, Liaoning 114001 China; 3 Department of Geriatrics, the Second Affiliated Hospital, Harbin Medical University, Harbin, Heilongjiang 150086 China; 4 Department of Pharmacology, Harbin Medical University, Harbin, 150081 China; 5 Hamon Center for Therapeutic Oncology Research, Department of Internal Medicine Pharmacology, University of Texas Southwestern Medical Center, Dallas, TX 75390 USA; 6 Division of Hypothalamic Research, Department of Internal Medicine, UT Southwestern Medical Center, Dallas, TX 75390 USA

## Abstract

Platelets play a multifaceted role in cancer progression and metastasis. Mean platelet volume (MPV) and platelet distribution width (PDW) are commonly used platelet parameters from routine blood test. The aim of the present study was to investigate the correlation between platelet indices and prognosis in patients with non-small cell lung cancer (NSCLC). A total of 270 patients who were diagnosed with NSCLC between January 2009 and December 2009 were retrospectively analyzed. Patients’ characteristics and hematologic tests data at initial diagnosis were collected. The overall survival rate was estimated using Kaplan-Meier method. The prognostic analysis was carried out with univariate and multivariate Cox regressions model. Reduced PDW was significantly correlated with T stage, N stage, TNM stage, and histological type of the disease. Moreover, survival analysis showed that the overall survival of patients with PDW ≥ 16.3% was significantly longer than that of those with PDW < 16.3% (*P* < 0.001). In multivariate Cox regression model, age, sex, TNM stage, and PDW were identified as independent prognostic factors for overall survival (for PDW, *P* < 0.001). In conclusion, reduced PDW is an unfavorable predictive factor of NSCLC patient survival. Further studies are warranted.

## Introduction

Lung cancer is one of the most common cancers worldwide, with non-small cell lung cancer (NSCLC) accounting for 80% of all diagnosed lung cancer cases. Current treatments, including surgery, radiotherapy, and chemotherapy, exhibit limited effectiveness for NSCLC. Moreover, long-term survival after surgical resection remains poor owing to the high rate of recurrence and metastasis^1^. Therefore, to improve treatment strategies, investigation of novel biomarkers is clinically urgently needed.

Platelets play a multifaceted role in cancer progression and metastases. Complex interactions between platelets and tumor cells result in tumor growth, aberrant angiogenesis, invasion, and metastasis^2, 3^. Several studies have reported that elevated platelets are associated with a poor prognosis in various types of cancer, including pancreatic cancer, gastric cancer, colorectal cancer, endometrial cancer, and ovarian cancer^4–8^. However, total platelet count is determined by the balance between the rate of production and consumption of platelets. A normal platelet count could conceal the presence of highly hypercoagulative and pro-inflammatory cancer phenotypes in the presence of efficient compensatory mechanisms^9^.

Mean platelet volume (MPV) is an indicator of activated platelets and is associated with different inflammatory conditions^10^. Platelet distribution width (PDW), another platelet index, indicates variation in platelet size and differentially diagnoses thrombocytopenia^11^. In addition, both MPV and PDW are easily detected with routinely used hemocytometers. A study found that reduced MPV predicted an unfavorable prognosis in patients with NSCLC^12^. However, PDW has not been studied completely.

The purpose of this study was to investigate the prognostic impact of the preoperative platelet indices on the survival in NSCLC patients.

## Results

The characteristics of the patients are summarized in Table 1. Overall, there were 177 (65.6%) male and 93 (34.4%) female patients, and the median age was 57.3 ± 9.3 years (range 32–80). The numbers of patients with adenocarcinoma, squamous cell carcinoma, and other carcinomas were 159, 103, and 8, respectively. In terms of the staging system, 84 cases were categorized as stage I, 146 as stage II, 30 as stage III and 10 as stage IV. Median follow-up time was 60.0 months. Throughout the follow-up period there were 51 deaths. The estimated cumulative 5-year survival for this patient population was 20.4% for OS.

**Table 1:** Baseline characteristics of the patients with lung cancer according to PDW levels.

Variables	Total n (%)	PDW ≤ 16.3 n (%)	PDW > 16.3n (%)	*P* value
Age (years)				0.495
≤60	174 (64.4)	27 (60.0)	147 (65.3)	
>60	96 (35.6)	18 (40.0)	78 (34.7)	
Gender				0.390
Male	177 (65.6)	32 (71.1)	145 (64.4)	
Female	93 (34.4)	13 (28.9)	80 (35.6)	
Smoking				0.476
Yes	151 (55.9)	23 (51.1)	128 (30.8)	
No	119 (44.1)	22 (48.9)	97 (43.1)	
Tumor size				0.078
>4 cm	84 (31.1)	9 (20.0)	75 (33.3)	
≤4 cm	186 (68.9)	36 (80.0)	150 (66.7)	
Differentiation				0.362
Well/moderate	195 (72.2)	30 (66.7)	165 (73.3)	
Poor	75 (27.8)	15 (33.3)	60 (26.7)	
Histological type				<0.001
Adenocarcinoma	159 (58.9)	26 (57.8)	133 (59.1)	
Squamous cell carcinoma	103 (38.1)	17 (37.8)	86 (38.2)	
Others	8 (3.0)	2 (4.4)	6 (2.7)	
T stage				<0.001
T1	84 (31.1)	19 (42.2)	65 (28.9)	
T2	146 (54.1)	18 (40.0)	128 (56.9)	
T3	30 (11.1)	8 (17.8)	22 (9.8)	
T4	10 (3.7)	0 (0)	10 (4.4)	
N stage				0.013
N0	180 (66.7)	31 (68.9)	149 (66.2)	
N1	34 (12.6)	3 (6.7)	31 (13.8)	
N2	51 (18.9)	10 (22.2)	41 (18.2)	
N3	5 (1.9)	1 (2.2)	4 (1.8)	
Cancer Stage				<0.001
I	143 (53.0)	18 (40.0)	125 (55.5)	
II	53 (19.6)	11 (24.4)	42 (18.7)	
III	74 (27.4)	16 (35.6)	58 (25.8)	

PDW, platelet distribution width.

The median value of PDW was 17.0 (range, 9.7–20.6). ROC curve analysis was used to determine the optimal cutoff value for PDW. ROC analysis showed that if the chosen cut-off point for PDW was 16.3, the specificity and sensitivity were 51.9% and 96.3%, respectively (AUC = 0.785, 95% CI: 0.732–0.833, *P* < 0.0001). Of the total of 270 patients, 45 patients (16.7%) were detected with PDW of less than or equal to 16.3, while there were 225 patients (83.3%) whose PDW was greater than 16.3.

Correlations between PDW and clinicopatholotic parameters are shown in Table 2. There were no significant differences in age, sex, BMI, smoking history, hemoglobin, platelet count, MPV, NLR, PLR, tumor size, and differentiation between the two groups. However, T stage, N stage, TNM stage, and histological type in two groups show significant difference.

**Table 2:** Baseline characteristics of the patients with lung cancer according to PDW levels.

Variables	PDW ≤ 16.3	PDW > 16.3	*P* value
Age (years)	57.6 (9.7)	57.2 (9.3)	0.786
BMI (kg/m^2^)	22.5 (2.8)	23.5 (3.1)	0.071
Hemoglobin (g/dl)	133.8 (18.7)	138.1 (18.6)	0.163
Platelet count (×10^9^/L)	267.6 (83.2)	241.5 (68.3)	0.053
MPV (fL)	8.8 (1.7)	8.6 (1.3)	0.435
NLR	2.97 (1.69)	2.49 (2.11)	0.152
PLR	151.5 (71.4)	138.2 (63.8)	0.214

PDW, platelet distribution width; BMI, body mass index; MPV, mean platelet volume; NLR, neutrophil-to-lymphocyte ratio; PLR, platelet-to-lymphocyte ratio.

**Figure 1:**
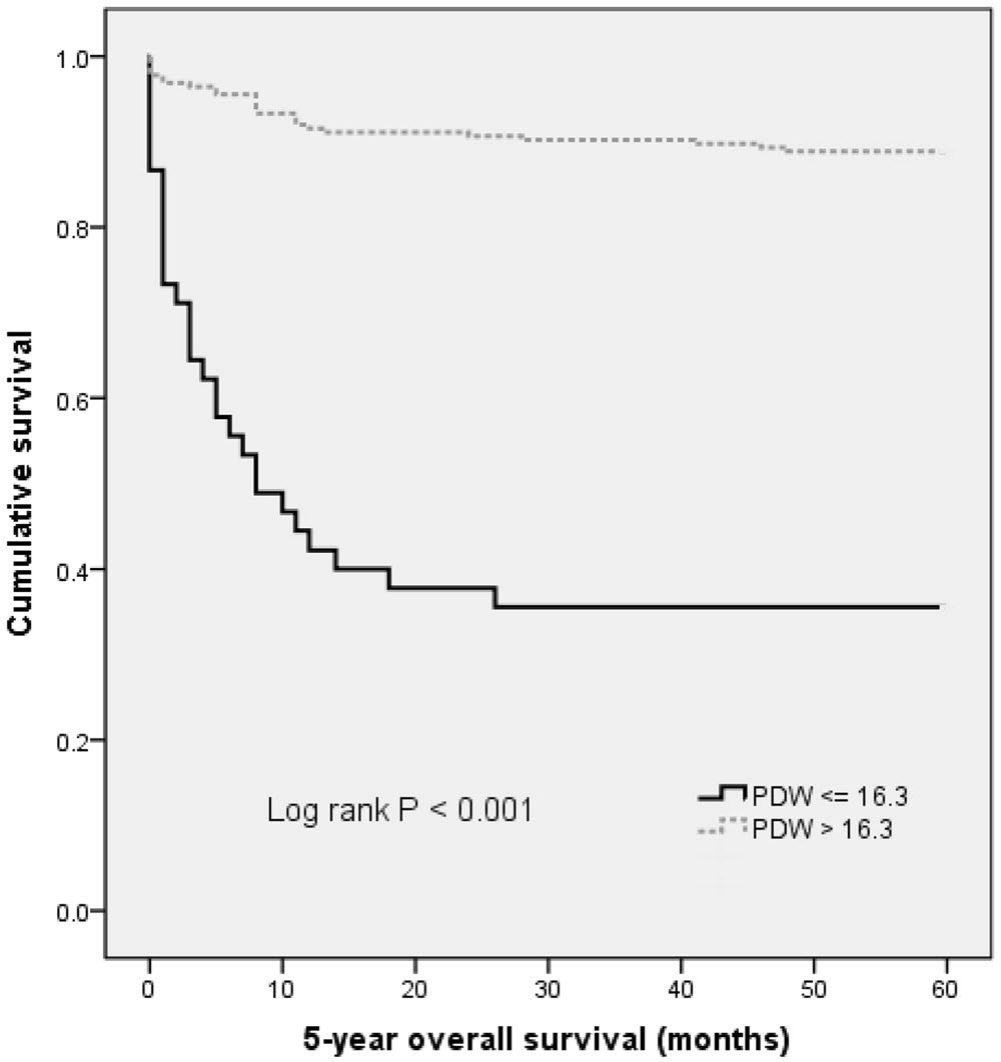
Kaplan–Meier analysis of overall survival in NSCLC patients.

The 5-year overall survival rate in this cohort was 20.4%. The 5-year overall survival rates in patients with PDW ≤ 16.3 and PDW > 16.3 were 35.6% and 88.9%, respectively. The overall survival of patients with PDW less than or equal to 16.3 was significantly shorter than those with PDW greater than 16.3 (*P* < 0.001) (Fig. 1).

The results of univariate and multivariate analysis were shown in Tables 3 and 4. Univariate analysis showed that age, sex, TNM stage, platelet count, PDW, NLR and PLR were significantly related to overall survival. Then, Cox regression analyzed models was constructed to compare the prognostic significance of PDW. Age, sex, TNM stage, and PDW were independent prognostic markers for overall survival. Notably, PDW before treatment was an independent factor for overall survival, with HR of 0.102 (95% CI: 0.058–0.180, *P* < 0.001).

**Table 3:** Univariate analysis of overall survival in patients with lung cancer.

	Hazard ratio	95% CI	*P*-value
Age (years) (≤60 versus >60)	1.725	1.010–2.946	0.046
Gender (male versus female)	2.470	1.243–4.909	0.010
BMI (kg/m^2^)	0.994	0.959–1.030	0.733
Smoking (yes versus no)	0.830	0.486–1.415	0.493
T stage	0.754	0.510–1.115	0.157
N stage	1.087	0.805–1.470	0.585
Cancer Stage (III versus I+II)	2.435	1.423–4.167	0.001
Tumor Size (cm) (≤4 versus >4)	0.934	0.521–1.676	0.820
Differentiation (well/moderate versus poor)	1.571	0.904–2.729	0.109
Hemoglobin (g/dl)	0.992	0.980–1.005	0.233
Platelet count (×10^9^/L)	1.004	1.001–1.008	0.020
MPV (fL)	1.140	0.949–1.370	0.162
PDW (%) (≤16.3 versus >16.3)	0.113	0.066–0.195	<0.001
NLR	1.117	1.041–1.200	0.002
PLR	1.004	1.002–1.007	0.003

PDW, platelet distribution width; BMI, body mass index; MPV, mean platelet volume; NLR, neutrophil-to-lymphocyte ratio; PLR, platelet-to-lymphocyte ratio.

**Table 4:** Multivariate analysis of overall survival in patients with lung cancer.

	Hazard ratio	95% CI	*P*-value
Age (years) (≤60 versus >60)	2.123	1.220–3.695	0.008
Gender (male versus female)	2.626	1.294–5.326	0.007
Cancer Stage (III versus I+II)	2.770	1.576–4.871	<0.001
Platelet count (×10^9^/L)	1.002	0.998–1.007	0.356
PDW (≤16.3 versus >16.3)	0.102	0.058–0.180	<0.001
NLR	1.096	0.929–1.294	0.277
PLR	1.000	0.994–1.007	0.988

Variables that showed a *P* value < 0.10 in univariate analysis were included in a multivariate Cox proportional hazards regression model.

## Discussion

With the present study, we demonstrated that reduced PDW was independently associated with the prognosis of NSCLC.

Despite best current medical and surgical treatment, the overall prognosis of patients with lung cancer remains poor. An increasing body of evidence point to the key roles of platelets in tumor progression^13^. Thrombocytosis is linked to with reduced survival in patients with various tumor types, including cancer of lung, ovary, endometrium, rectum, kidney, stomach, pancreas, brain, and breast. Recent studies revealed that cancer-associated thrombocytosis was a paraneoplastic phenomenon. Tumors could promote platelet production and activation through the interleukin (IL)-6 pathway^14^. In lung cancer, platelet-derived growth factor (PDGF) beta-receptor expression was found to be significantly higher in rare sarcomatoid NSCLC versus non-sarcomatoid NSCLC controls^15^.

A specific mechanism to explain reduced PDW in lung cancer remains to be determined. Cancer-associated inflammation may be a common mechanism for decreased PDW. PDW is a measure of platelet volume heterogeneity. Platelet volume is determined both during megakaryopoiesis and during thrombopoiesis. Megakaryocytic maturation, platelet production and platelet size could be modulated by cytokines, such as interleukin-6 (IL-6), granulocytes colony stimulating factor (G-CSF) and macrophage colony stimulating factor (M-CSF)^16^. Elevated interleukin-6 (IL-6) has been observed in almost all types of tumors acting as a major pro-inflammatory mediator in tumor microenvironment. Numerous studies suggests that IL-6 promotes tumorigenesis by regulating apoptosis, survival, proliferation, angiogenesis, metastasis and metabolism^17^. Moreover, megakaryopoiesis and subsequent thrombopoiesis in cancer may be stimulated by pro-inflammatory cytokines G-CSF and M-CSF, which could be secreted by tumor cells^18^. In accord with previous results, we found that reduced PDW is negatively correlated with white blood cell count (see Supplementary Table 1).

However, the change of PDW in different types of cancers is inconsistent. PDW has been noted to be increased in gastric cancer and lung cancer^19, 20^. In contrast, PDW is decreased in thyroid cancer and breast cancer^21, 22^. The conflicting data maybe due to small sample sizes, failure to rule out confounding factors, different tumor type, and selected populations. Therefore, further research on PDW levels in cancer is warranted.

The present study has some limitations. Firstly, this was a single-center retrospective study and multicentric prospective studies are needed to reduce selection bias. Secondly, a mechanistic explanation for our findings is not provided by our data and further study is needed to identify the precise mechanism. Thirdly, because the study sample includes only Chinese participants, caution is needed in extrapolating our results to different ethnic groups.

In conclusion, our study indicates that a preoperative reduced PDW is an independent prognostic biomarker for overall survival in NSCLC that is superior to NLR and PLR. The results suggest that platelets may play a greater role in promoting NSCLC progression. Additional investigations are needed to fully understand the potential mechanism.

## Materials and Methods

### Study population

The records of patients with lung cancer who were admitted to Harbin Medical University Cancer Hospital, Harbin Medical University between January 2009 and December 2009 were retrospectively reviewed. Patients meeting all of the following requirements were eligible for enrollment: (1) undergone complete surgical resection and diagnosis of lung cancer was confirmed by histology; (2) without distant metastasis at diagnosis; (3) untreated before diagnosis. Exclusion criteria included: hematological disorders, autoimmune diseases, systemic inflammatory diseases, coronary artery disease, hypertension, diabetes mellitus, thyroid disease, renal disease, hepatic disorder and other cancer, and medical treatment with anticoagulant, statins, and acetylic salicylic acid. The pathological stages of patients were determined according to the international TNM classification system for lung cancer^23^.

Written informed consents were obtained from all patients. This study was approved by the Institutional Review Board of Harbin Medical University Cancer Hospital of Harbin Medical University. All studies were conducted according to guidelines (Declaration of Helsinki) for biomedical research. The last follow-up date was December 31, 2014. Overall survival (OS) was calculated from the date of surgery to the date of death or last follow-up.

### Clinical examination and biochemical measurements

All the subjects underwent physical examination. Body mass index (BMI) was calculated as the ratio of weight (kg) to height squared (m^2^). Clinical data including smoking status, medical history and medication use were recorded for each subject. Venous blood samples after a 10-hour overnight fasting were collected from the individuals within 1 week prior to surgery. White blood cell (WBC), haemoglobin, and platelet indices were measured by an autoanalyzer (Sysmex XE-2100, Kobe, Japan). The whole blood samples were collected in EDTA-containing tubes, and all samples were processed within 30 minutes after blood collection. The inter- and intra-assays coefficients of variation (CVs) of all these assays were below 5%. The platelet-to-lymphocyte ratio (PLR) was calculated as the absolute platelet count measured in ×10^9^/L divided by the absolute lymphocyte count measured in ×10^9^/L. The neutrophil-to-lymphocyte ratio (NLR) was calculated as the absolute neutrophil count measured in ×10^9^/L divided by the absolute lymphocyte count measured in ×10^9^/L. The ideal cutoff value for PDW was determined applying receiver operating curve analysis.

### Statistical analysis

All statistical analyses were performed using SPSS Statistics version 22.0 (SPSS Inc., Chicago, IL, USA). The descriptive statistics are presented as means ± SD or medians (interquartile range) for continuous variables and percentages of the number for categorical variables. When baseline characteristics between two groups were compared, normally distributed continuous variables were compared with the Student t test and skewed-distributed with the Mann-Whitney U test. The Chi-square test was used for categorical variables. Survival curves were visualized by the Kaplan–Meier method and examined by a log-rank test. Univariate and multivariate analyses of the significance of demographic and clinico–pathological parameters associated with lung cancer were assessed by a Cox proportional hazards regression model. Variables that showed a *P* value < 0.1 in univariate analysis were included in a multivariate Cox proportional hazards regression model. The significance level was set at *P* < 0.05.

## Electronic supplementary material

Supplementary Information
