# Supplementary material for: Platelet distribution width correlates with prognosis of non-small cell lung cancer
Source: Sci Rep. 2017 Jun 14;7:3456. doi: 10.1038/s41598-017-03772-z (PMC5471191; doi:10.1038/s41598-017-03772-z)
Supplement: Supplementary file 1 — Supplementary Information [file 41598_2017_3772_MOESM1_ESM.pdf]

Platelet distribution width correlates with prognosis of non-small cell lung cancer

running heading: PDW and prognosis in NSCLC

Ming-ming Cui[1#]; Na Li[1#]; Xing Liu[2#], Zhi-yuan Yun[1]; Ye Niu[3]; Yong Zhang[4]; Boning Gao[5]; Tiemin Liu[1,6]; Rui-tao Wang[1,*].

[1]Department of Internal Medicine, Harbin Medical University Cancer Hospital, Harbin Medical University, Harbin, Heilongjiang, 150081, China

[2]Department of Endocrinology, Ansteel Group Hospital, AnShan, Liaoning, 114001, China

[3]Department of Geriatrics, the Second Affiliated Hospital, Harbin Medical University, Harbin, Heilongjiang, 150086, China

[4]Department of Pharmacology, Harbin Medical University, Harbin, 150081, China

[5]Hamon Center for Therapeutic Oncology Research, Department of Internal Medicine Pharmacology, University of Texas Southwestern Medical Center, Dallas, TX 75390, USA

[6]Division of Hypothalamic Research, Department of Internal Medicine, UT Southwestern Medical Center, Dallas, TX, 75390, USA

[*]To whom correspondence should be addressed: Rui-tao Wang. Department of Internal Medicine, Harbin Medical University Cancer Hospital, Harbin Medical University, NO.150 Haping ST, Nangang District, Harbin, 150081, China; Tel: 86-451-86298352; Fax: 86-451-86298353; Email: ruitaowang@126.com.

Ming-ming Cui, Na Li, and Xing Liu contributed equally to this work.

word count: 1798;   the number of figures and tables: 5

Supplementary Table 1

Correlation coefficient (r) for PDW in relation to WBC, neutrophil, platelet count, and MPV in NSCLC.

	WBC		Neutrophil		Platelet count		MPV	
	r	p	r	p	r	p	r	p
PDW	-0.137	0.024	-0.156	0.010	-0.211	<0.001	0.073	0.229
